# Understanding the development of teachers’ self-efficacy to promote self-regulated learning: a quasi-experimental study on the role of experience, mindset, and self-regulated learning skills

**DOI:** 10.1007/s10212-025-00996-w

**Published:** 2025-08-27

**Authors:** Johannes Jud, Carmen Nadja Hirt, Tabea Daria Eberli, Amina Rosenthal, Yves Karlen

**Affiliations:** Institute of Education, University of Zurich, Kantonsschulsstrasse 3, CH-8001 Zurich, Switzerland

**Keywords:** Self-regulated learning, Teacher self-efficacy, Mindsets, Teachers’ experience

## Abstract

Teachers’ self-efficacy in promoting self-regulated learning (TSE-SRL) is pivotal for self-regulated learning (SRL) practices. This study investigated the effects of mastery and vicarious experiences as sources of self-efficacy within a professional development (PD) program on TSE-SRL. Additionally, the moderating role of teachers’ prior SRL experience, SRL mindsets and their own SRL skills was examined. The sample included fifty-four lower secondary school teachers who participated in a quasi-experimental PD program with an experimental group (*n* = 31) and a control group (*n* = 23). Both groups were exposed to mastery and vicarious experiences. However, while the experimental group focused on developing competencies for promoting SRL, the control group focused on teachers’ competencies to promote social skills. Results from several regression models revealed that TSE-SRL was developed through the PD program for both groups, with a slightly higher improvement in the experimental group. Teachers’ previous experience was the only variable moderating this effect. The study provides information about the importance of mastery and vicarious experiences for TSE-SRL and the design of PD programs to foster TSE-SRL.

## Introduction

Skills in self-regulated learning (SRL) are essential for academic achievement and lifelong learning and are crucial for overcoming current and future challenges (Dent & Koenka, 2016; Karlen & Hertel, 2024). SRL skills can be fostered by teachers (De Boer et al., 2018), but research has shown that teachers rarely do so (Rosenthal et al., 2024). Findings about teachers’ professional competencies indicate that teachers’ self-efficacy to promote SRL (TSE-SRL) is one of the most important predictors of their SRL promotion (Chatzistamatiou et al., 2014; Karlen et al., 2020). TSE-SRL is also indirectly linked to students’ SRL motivation and SRL skills (Karlen et al., 2024; Jud et al., 2024). Therefore, it is important to understand how TSE-SRL can be fostered.

The situated expectancy value theory (SEVT) (Eccles & Wigfield, 2020) and Bandura's social cognitive theory (1997) highlight that one’s experiences, such as mastery and vicarious experiences, are important sources for developing self-efficacy. Both theories also outline individual factors that shape how these experiences are interpreted. This includes previous experience in a task, one’s beliefs or mindsets (Dweck, 2016; Eccles & Wigfield, 2020), and the ability to use adaptive strategies to regulate motivational and cognitive responses to experiences (Morris et al., 2017). While Morris et al. (2017) refer to these as coping strategies, they are conceptually aligned with SRL skills, such as motivational and metacognitive regulation (Schraw et al., 2006; Wolters, 2011).

Meta-analyses have shown that interventions, such as professional development (PD) programs incorporating mastery and vicarious experiences as sources of self-efficacy, can positively impact the development of teachers'self-efficacy (TSE). Additionally, different aspects, including reflective elements, moderate this effect (Mok et al., 2023; Täschner et al., 2024). However, self-efficacy is considered a domain-specific construct (Tschannen-Moran et al., 1998; Usher, 2023). In the context of SRL, few ambiguous findings exist about whether TSE-SRL develops within PD programs (Dignath, 2021; Heirweg et al., 2021). Further, these studies mostly didn’t explicitly refer to mastery and vicarious experiences and provided hardly any insights about possible moderating factors.

This study, therefore, aims to investigate the development of TSE-SRL in two ways: First, it analyses the effect of an SRL PD program, fostering mastery and vicarious experiences, on the development of TSE-SRL. Second, the study aims to investigate how this potential effect on the development of TSE-SRL is moderated by teachers’ characteristics, including their previous level of experience in promoting SRL, their SRL mindsets, and their own SRL skills. In line with research on self-efficacy development (e.g., Morris et al., 2017), such skills include the ability to regulate one’s own learning and to cope with experiences adaptively. Therefore, this study provides a deeper understanding of TSE-SRL development within PD programs.

### Self-regulated learning and its promotion

Although different definitions and models of SRL exist, there is an overall agreement that SRL refers to learners as active agents of their learning process who activate their strategy knowledge about strategies, effectively use various strategies to regulate different aspects of their learning, and stay motivated to achieve their goals (Pintrich, 2000). Therefore, successful SRL requires a combination of cognitive, motivational, and metacognitive skills, including the ability to use different metacognitive, motivational, emotional, and cognitive strategies (Pressley et al., 1987; Schraw et al., 2006). SRL skills can be developed, but this development requires various steps, including learners observing models, emulating this SRL-related behaviour, and practising SRL until they can finally adapt and perform SRL in different situations (Zimmerman, 2002). Therefore, to successfully develop SRL skills, guidance is required. Teachers play an important role in supporting their students’ development of SRL skills. This includes various tasks and roles, which change according to students’ development of SRL skills (e.g., Karlen & Hertel, 2024). Students with low SRL skills require more explicit strategy instructions from their teachers. When students have gained more SRL skills, more implicit and indirect promotion, such as learning environments that allow students to apply strategies and take responsibility for their learning, become relevant (Karlen & Hertel, 2024; Pressley & Harris, 2006; Zimmerman, 2000). However, research has shown that teachers mainly support SRL indirectly and rarely support students’ SRL development through direct modelling and explaining strategies (Dignath & Büttner, 2018; Kistner et al., 2010; Spruce & Bol, 2015; Rosenthal et al., 2024). TSE-SRL has been identified as a key predictor of whether and how teachers engage in SRL-supportive practices (Chatzistamatiou et al., 2014; Dignath-van Ewijk, 2016; Jud et al., 2023). Therefore, it is crucial to better understand how teachers’ TSE-SRL develops.

### Teachers’ self-efficacy to promote SRL

Self-efficacy is grounded in Bandura’s socio-cognitive theory and is defined as “beliefs in one’s capabilities to organise and execute the course of action required to produce given attainments.” (Bandura, 1997, p.3). Additionally, SEVT posits that one’s expectancies of success, closely related and often assessed through the construct of self-efficacy, are situated alongside values as a central predictor of choices and performance. The importance of teachers’ self-efficacy (TSE) for teaching quality, their psychological well-being, and their students’ achievements has been shown in different studies (e.g. Zee & Koomen, 2016).

However, TSE is considered a domain-specific construct, and therefore, various self-efficacy scales exist (Tschannen-Moran et al., 1998; Usher & Pajares, 2008; Weber et al., 2019). Moreover, findings indicate that different teachers’ self-efficacy in different teaching domains relates differently to outcomes and malleability due to interventions (Chen et al., 2020). SRL is understood as a distinct instructional domain that needs deep understanding and knowledge about its promotion (De Smul et al., 2018; James & McCormick, 2009; Karlen & Hertel, 2024). Therefore, teachers can have different perceptions of self-efficacy regarding domains of teaching a subject matter and teaching SRL (TSE-SRL). De Smul et al. (2018) developed a specific SRL scale that measures teachers’ perceived ability to integrate various activities promoting SRL in their daily classroom practices. The authors showed that TSE-SRL, compared to TSE, is particularly relevant to teachers’ SRL promotion. Additionally, multi-level analyses demonstrated that TSE-SRL is relevant to students’ SRL outcomes, such as their SRL skills (Heirweg et al., 2020) and SRL motivation (Jud et al., 2024).

### Developing teachers’ self-efficacy in professional development programs

SEVT highlights that competency beliefs, such as self-efficacy, are dynamic and change over time, and one’s experience is fundamental for this change. TSE is assumed to influence choices and performance, which in turn lead to certain experiences. These experiences, in turn, are interpreted and influence inferences drawn about one’s competencies in a task, affecting one’s self-efficacy regarding future tasks (Eccles & Wigfield, 2020). Although Bandura (1997) described self-efficacy as relatively stable once developed, he also emphasised its malleability through mastery and vicarious experiences in the context of new or challenging tasks. This view aligns with SEVT’s emphasis on the dynamic nature of motivational beliefs, as both frameworks recognise that experiences within a specific context can shape future self-efficacy. First, *mastery experiences* foster one’s self-efficacy through past attainments. People achieving goals through direct, personal actions experience efforts and success and are more likely to approach similar tasks confidently. In the context of teaching, mastery experience has been defined as the “enactment of teaching skills in an authentic teaching situation” (Mok et al., 2023, p.1). Second, *vicarious experiences* are derived when individuals observe a model performing a task of interest. These experiences may be profound for one’s self-efficacy when a task is relatively new and when the model or the compared group is perceived as like oneself. In the context of SRL, teachers’ self-efficacy could be influenced by targeted mastery and vicarious experiences, especially when SRL promotion is not yet an established part of their teaching repertoire.

Various studies exist about how TSE develops through targeted interventions such as PD programs. A recent meta-analysis revealed that interventions, including different sources of self-efficacy, can effectively promote TSE (Täschner et al., 2024). The effects of the different sources may be intertwined in the intervention, and all sources may be necessary for an overall effect (Bach, 2022; Täschner et al., 2024). However, mastery experiences are assumed to be most influential for self-efficacy, especially when a task is highly demanding (Bandura, 1997), such as promoting SRL (Hirt et al., 2022). Accordingly, when comparing interventions that included only one source of self-efficacy, interventions with vicarious or mastery experiences showed higher effects than social persuasions (Täschner et al., 2024).

In the context of SRL, there are ambiguous results regarding the effect of PD programs as interventions for developing TSE-SRL. While some studies found no significant improvement in teachers’ self-efficacy (e.g., Heirweg et al., 2021), others did (e.g., Cleary et al., 2022; Dignath, 2021; Finsterwald et al., 2013). However, these studies primarily focused on whether an increase in TSE-SRL occurred, without explaining this development based on theoretical assumptions about self-efficacy and including moderator analysis. In particular, most studies did not explicitly refer to Bandura’s sources of self-efficacy, such as mastery experiences or vicarious experiences. Nevertheless, such sources can often be identified within the descriptions of the PD programs. For instance, many PD programs included elements such as modelling of SRL-promoting practices and opportunities for peer observation or exchange, which can be interpreted as providing vicarious experiences or mastery experiences through practical application (Hirt et al., 2025). Heirweg et al. (2021) explained the missing effects in their study by the duration of their PD program: their one-year PD program might have been too short for teachers to develop their TSE-SRL due to the mastery experiences based on their SRL promotion. However, this implicitly leads to the conclusion that mastery experience and the interpretation processes of these experiences are crucial for the development of TSE-SRL, and vicarious experiences or social persuasion alone are not enough to develop TSE-SRL.

### Moderating factors of self-efficacy development within a professional development program

The development of self-efficacy is assumed to be moderated by different constructs (Bandura, 1997; Eccles & Wigfield, 2020; Morris et al., 2017). First, the effect of the sources on self-efficacy is conceived to differ depending on someone’s previous experience level regarding a task. For instance, the effect of vicarious experiences is assumed to be higher when individuals are confronted with new tasks or have hardly any previous experience (Bandura, 1997). Accordingly, self-efficacy is assumed to be relatively stable when individuals have more experience with a task. Thus, more experienced teachers are assumed to show lower changes in TSE within interventions than teachers with less experience (Täschner et al., 2024). However, research about the influence of teachers’ experience on the development of self-efficacy is mixed. While some report TSE as a relatively stable construct in later career stages (Savolainen et al., 2022), a meta-analysis showed that the development of TSE does not differ between pre- and in-service teachers (Täschner et al., 2024). This effect was explained by the fact that teachers are likely to overestimate their TSE for new tasks, and a shift in this perception can be expected when they are exposed to task (Pfitzner-Eden, 2016; Täschner et al., 2024). In the context of SRL, studies indicate that teachers’ experience in promoting SRL is related to their SRL promotion and their TSE-SRL (e.g. Hirt et al., 2022; Jud et al., 2023). However, these studies do not indicate how teachers’ experiences influence their TSE-SRL development. One of the few studies pointing in this direction found that teachers with initially high competence profiles in promoting SRL increased their TSE-SRL more than teachers with lower initial competences in SRL promotion (Dignath, 2021). The author concluded that teachers already highly competent in SRL promotion would benefit more from short-term PD programs in terms of developing their TSE-SRL.

Second, next to teachers’ level of experience, the effect of the sources of self-efficacy is conceptualised to be moderated by how individuals process and interpret their experiences (Bandura, 1997; Eccles & Wigfield, 2020; Morris et al., 2017). Information from various sources must be perceived, selected, and incorporated into individuals’ judgments to influence the development of self-efficacy. One’s mindsets about the malleability of abilities are assumed to influence how information about the sources of self-efficacy is interpreted and, thus, influence the development of self-efficacy (Chen & Usher, 2013). Individuals with fixed mindsets believe that certain abilities, such as SRL skills, cannot be changed, while individuals with growth mindsets believe that abilities are malleable (Dweck, 2016). In their meta-analysis, Bardach et al. (2024) showed that teachers’ growth mindset predicts TSE. In the context of SRL, individuals might either assume that SRL abilities are relatively stable and cannot be developed through training (fixed mindset), or they assume that SRL abilities are malleable and can be improved through practice (growth mindset) (Hertel & Karlen, 2021). These mindsets might, for example, affect teachers’ interpretation of mastery experiences: Teachers who assume that SRL skills can be developed through training might attribute students’ development of SRL skills to their SRL promotion and, therefore, feel more self-efficacious in doing so. Regarding vicarious experiences, Bandura (1997) emphasised that the impact of observing others is stronger when the observer perceives the model as similar to themselves and believes that they are capable of performing the observed behaviour. Applied to SRL, this suggests that teachers may benefit more from vicarious experiences when they believe that the skills required for SRL promotion are learnable and applicable to their own teaching context. Thus, teachers who observe SRL promotion might develop their TSE-SRL more strongly when they hold growth mindsets about SRL. Evidence suggests that students with a growth mindset draw on multiple sources of self-efficacy, whereas those with a fixed mindset rely on fewer sources and are less likely to develop self-efficacy (Chen & Usher, 2013). Regarding teachers’ mindsets about SRL, Cleary et al. (2022) found that teachers who focused on internal, controllable factors to improve SRL benefited more from their teacher training than teachers who focused on external or uncontrollable factors.

Third, teachers’ own SRL skills are assumed to moderate the relationship between sources of self-efficacy and TSE development. For instance, teachers’ strategies to cope with negative information resulting from experiences could lower the negative effects of these experiences on self-efficacy (Shen, 2009). These strategies correspond to motivational regulation strategies, which are part of motivational skills within SRL and can be defined as one’s efforts to influence or control motivation (Wolters, 2011). These strategies also include ways to regulate one’s self-efficacy (Wolters, 2011). However, teachers must be aware of these strategies and value their use to successfully employ motivational strategies in enhancing the sources of self-efficacy (Morris et al., 2017. Next to motivational strategies, reflection on what individuals experience might influence the development of their self-efficacy (Usher, 2023). Empirically, moments of reflection within interventions are important for developing self-efficacy (Täschner et al., 2024). However, reflection is a metacognitive skill and, as such, part of one’s SRL (Schraw et al., 2006). Therefore, teachers may need these skills to process the moments of reflection after a particular experience. Teachers’ own SRL skills, including their metacognitive and motivational skills, have been conceptualised as core professional competencies (Karlen et al., 2020) and linked to TSE-SRL (Karlen et al., 2023b). Teachers who understand their own learning and reflect upon their experiences are assumed to create learning contexts that foster students’ SRL skills (Paris & Winograd, 2003). However, based on cross-sectional data, no conclusions can be drawn about the influence of teachers’ own SRL skills on the development of TSE-SRL.

### Present study

**Fig. 1:**
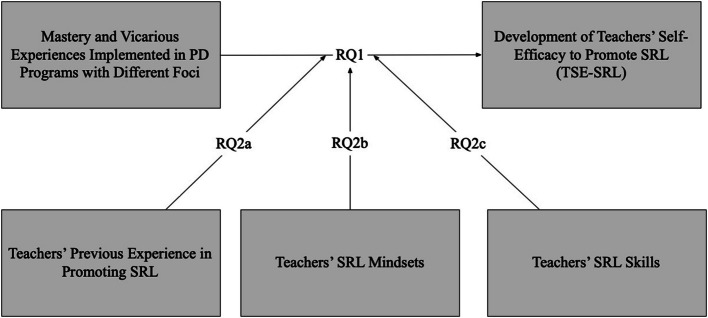
Overview of the Central Constructs and Research Questions (RQ)

In the context of SRL, findings about the development of TSE-SRL within a PD program are ambiguous, and few explicit theoretical references to experiences as sources of self-efficacy can be found. Additionally, there are hardly any results available regarding teachers’ characteristics, which moderate the effect of mastery and vicarious experiences on the development of TSE-SRL. Therefore, this study aims to gain a deeper understanding of the development of TSE-SRL through a quasi-experimental research design. The influence of mastery and vicarious experiences in two PD programs with different focus on the development of TSE-SRL was examined, as well as the moderators that may impact this effect. The following research questions (RQ) are addressed, which are shown in Fig. 1:

- *RQ1:* How does an SRL PD program, which includes mastery and vicarious experiences, affect the development of teachers’ self-efficacy in promoting SRL compared to an alternative PD program?
- Hypothesis 1 [H1]: As theoretically assumed (Bandura, 1997; Morris et al., 2017) and empirically tested in recent meta-studies (e.g. Täschner et al., 2024), we expect that the PD program, including the promotion of mastery and vicarious experiences, positively relates to teachers’ development of TSE-SRL. Therefore, we expected that teachers in the EG would how higher development in TSE-SRL than teachers in the CG.
- *RQ2:* Which individual factors, including teachers’ previous experiences in promoting SRL (RQ2a), teachers’ SRL mindsets (RQ2b) and teachers’ SRL skills (RQ2c), moderate the effect of the PD program on the development of TSE-SRL?
- *Hypothesis [H2a]:* The effect of the experiences is assumed to be especially high when somebody has relatively little previous experience in a specific domain (Bandura, 1997). Therefore, we hypothesise that teachers’ experience in promoting SRL would negatively moderate the effect of the experiences within the PD program.
- *Hypothesis [H2b]:* Regarding teachers’ SRL mindsets, based on the theoretical assumed effect of one’s interpretation of experiences (Bandura, 1997) and previous findings in the context of SRL (Cleary et al., 2022; Hirt et al., 2022), we assumed that teachers’ SRL mindsets would positively moderate the effect of mastery and vicarious experiences on the development of TSE-SRL.
- *Hypothesis [H2c]:* Finally, regarding teachers’ own SRL skills, we hypothesised that these skills would positively moderate the effect of mastery and vicarious experiences. Theoretical models and meta-analyses suggest that individuals’ ability to apply adaptive cognitive and motivational strategies, as well as to engage in reflection, can influence how they interpret and respond to efficacy-related experiences (Morris et al., 2017). These strategies, although not explicitly framed as SRL skills, are conceptually related to components of SRL, such as motivational regulation and metacognitive reflection (Schraw et al., 2006; Wolters, 2011).

## Method

### Participants

This study used data from teachers enrolled in a PD program with a quasi-experimental control group design. *n* = 54 teachers teaching different subjects participated in the study. Teachers were, on average, *M* = 34.65 years old (*SD* = 8.95). 61.1% were female and had an average general professional experience of *M* = 16.48 years (*SD* = 10.13). The experimental group (EG) consisted of *n* = 31 teachers (31.6% female), and the control group (CG) of *N* = 23 teachers (65.2% female). Results from t-tests revealed that no significant differences existed between the groups in terms of age (*M*_EG_ = 35.21, *SD* = 8.35; *M*_CG_ = 33.50, *SD* = 9.85; t(51) =.921, *p* =.362) and general professional experience (*M*_EG_ = 15.39, *SD* = 9.69; *M*_CG_ = 17.96, *SD* = 10.73; t(52) = -.722, *p* =.474).

### Procedure

The PD program lasted one school year (2021/2022) and included five training days throughout the year. The program was designed for lower secondary teachers and promoted the implementation of SRL promotion in everyday classrooms. While the EG focused on teachers’ competences to promote SRL, the CG focused on promoting social competencies. Through self-selection, the teachers were allocated to the EG and CG (Shadish et al., 2002). The training took place in university locations in three different cities. When signing up for the PD program, they could select between available locations for attendance without knowing which location referred to the CG or EG. Participation in the PD program was voluntary, and the enrolled teachers were informed and consented to the scientific monitoring. All research procedures met the ethical standards of the Swiss Science Foundation. The study received approval from the university’s Ethics Committee.

### Instruments

#### Mastery and vicarious experiences as sources of self-efficacy within the PD program

Teachers were coded corresponding to their group to assess the effect of mastery and vicarious experiences as sources of TSE-SRL (CG = 0; EG = 1). Both PD programs included several measures to foster teachers’ mastery and vicarious experiences (for a detailed program of the PD program, see Hirt et al., 2025). However, the focus differed: While the PD program in the EG focused on promoting TSE-SRL, the PD program in the CG focused on promoting teachers’ self-efficacy to promote social competencies.

In the EG, to enable mastery experiences, the teachers were introduced on how to directly and indirectly promote different SRL components. Additionally, different materials, including teaching materials to promote different SRL skills, templates to diagnose and assess their students’ SRL skills, and examples of lesson plans were handed to the teachers. Teachers were then given a choice between different tasks on how to apply the provided information and materials in their classes. Implementing specific tasks is important as actual practical experiences and not only encouragement to do so are crucial for mastery experiences (Täschner et al., 2024). Second, to enhance teachers’ vicarious experiences within the PD program, examples of SRL promotion were shown, including a video of teachers performing SRL promotion in lower secondary classes. Models in a comparable situation to oneself are supposed to strengthen the effect of vicarious experience. Additionally, teachers presented their work at the beginning of each following session, which also enabled vicarious experiences for the EG participants as they could see other teachers’ models of promoting SRL in their classrooms. Although these presentations may also have included some form of social persuasion (e.g., informal feedback about the work done), elements of social persuasion were not included in the PD program as a formal aspect. Finally, researchers with expertise in SRL and previous teaching experience executed the PD program. The experience of being a teacher is important because credibility and relatedness to the group are assumed to enhance the impact of various sources of self-efficacy (Bandura, 1997). Although the PD program for the CG included similar measures to promote teachers’ self-efficacy (instructions on how to promote social competencies, provision of teaching materials, and tasks on how to apply the promotion in class), these measures clearly focused on promoting social competencies rather than SRL.

#### Other measures

The instruments for all measures were assessed with online questionnaires before and after the PD program. Table 1 presents the descriptive statistics and internal consistencies.

**Table 1:** Descriptive statistics, internal consistencies, and intercorrelations

Variables	1	2	3	4	5	6	7	8
1. Mastery and vicarious experiences focus in PD program	-							
2. TSE-SRL t1	−0.03	-						
3. TSE-SRL t2	0.18	0.49^**^	-					
4. Development of TSE-SRL t2-t1	0.27^†^	0.00	0.87^**^	-				
5. Previous experience in promoting SRL t1	−0.07	0.59^**^	0.29^*^	−0.03	-			
6. SRL mindsets t1	−0.07	0.09	−0.11	−0.20	0.08	-		
7. Own SRL skills t1	0.02	0.31^*^	0.27	0.11	0.08	0.29^*^	-	
8. Teaching experience t1	−0.13	0.11	0.22	0.19	0.44^**^	0.22	−0.09	-
α	-	0.85	0.78	-	-	0.68	0.83	-
Possible range	0/1	1–6	1–6	-	1–4	1–6	1–6	0–45
*N*_*total*_	54	53	49	48	54	54	54	54
*N*_*EG*_	31	30	28	27	31	31	31	31
*N*_*CG*_	23	23	21	21	23	23	23	23
*M*_*total*_	-	3.46	3.96	0.00	2.33	5.40	4.61	16.48
*M*_*EG*_	-	3.43	4.07	0.14	2.29	5.36	4.70	15.39
*M*_*CG*_	-	3.48	3.82	−0.17	2.39	5.44	4.47	17.96
*SD*_*total*_	-	0.92	0.69	0.60	0.75	0.57	0.54	10.13
*SD*_*EG*_	-	1.04	0.67	0.56	0.73	0.56	0.55	9.61
*SD*_*CG*_	-	0.75	0.69	0.61	0.78	0.58	0.52	10.72

*EG* = experimental group, *CG* = control group

^†^*p* <.10. ^*^p <.05. ^**^p <.01

*TSE-SRL:* We used Hirt et al.’s scale (2022) to assess teachers’ self-efficacy regarding SRL promotion. We included four items (example item: “How well can you inform your students about the importance and usefulness of self-regulated learning strategies?”). The answers were provided on a scale from 1 (I cannot do that at all) to 6 (I can do that very well).

*Previous experience in SRL promotion*. The previous experience in SRL promotion was measured by one item: «How much experience do you have in promoting self-organised learning in the classroom?» The answers were provided on a scale of 1 ((almost) no experience) to 4 (very much experience).

*SRL Mindsets.* Teachers’ *SRL* mindsets were assessed using a validated scale from Hertel and Karlen (2021). The scale included three items (example item: “Self-regulated learning…(1) cannot be improved by practice/(6) can be improved by practice.”). Higher scores meant that teachers have a growth rather than a fixed mindset about SRL.

*Own SRL Skills*. We used two subscales from the Karlen et al.’s (2023b) scale to assess teachers’ SRL skills. This includes one scale to assess teachers’ metacognitive skills consisting of five items (example item: “I can evaluate well whether I am achieving my goals with the chosen actions.”) and one scale to assess teachers’ motivational skills with four items (example item: “When my stamina for learning wanes, I can still motivate myself to learn again.”). The answers for the scales were rated on a six-point scale from 1 (does not apply at all) to 6 (entirely true). Karlen et al. (2023b) conducted an EFA to explore the factor structure and a subsequent CFA to test a second-order model, in which a single higher-order latent SRL skill factor demonstrated good model fit.

*Control Variable.* As teachers may react differently to the same interventions on self-efficacy across different career stages (Van Der Scheer & Visscher, 2016), we included teachers’ general teaching experiences as a control variable (“How many years of professional experience do you have?”).

### Data analysis

**Fig. 2:**
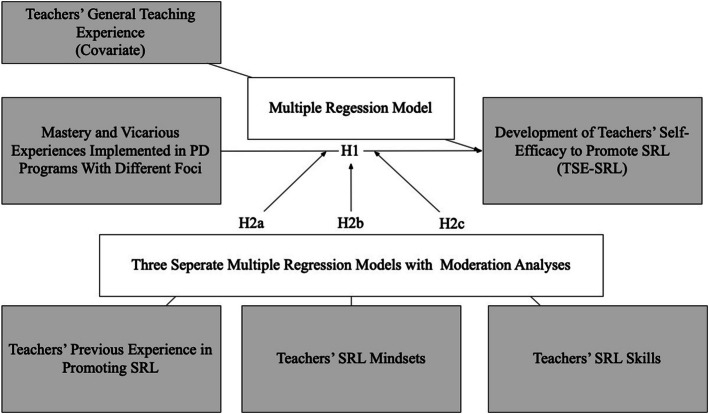
Overview of the data analyses and the hypotheses (H)

First, we used IBM SPSS Statistics Version 29 to execute a linear regression analysis between teachers’ self-efficacy for SRL at the first and second measurements. The resulting residual change variable was then used to represent the development of TSE-SRL within the PD program. This procedure enables the control of differences in self-efficacy levels between the EG and CG at the beginning of the PD program (Twist & Proper, 2005) To ensure comparability between the EG and the CG, independent-samples t-tests were conducted on all key variables. An overview of the data analysis is provided in Fig. 2.

H1: We conducted a multiple regression analysis using the lm function from the stats package in R (version 4.3.1). In this model, we regressed the development of TSE-SRL (residual change score) on group affiliation (coded as experimental group = 1, control group = 0), representing the differential content focus of the PD program (SRL skills vs. social skills). Teachers’ general professional experience was included as a control variable.

H2a-2c: Due to the limited sample size, we conducted three separate moderation analyses to test whether the three constructs would moderate the effect of mastery and vicarious experiences as sources of self-efficacy. Therefore, we included the interaction term between the residual change variable and one variable assumed to moderate this effect (Models 2b, 3b, 4b). Additionally, we conducted multiple regression models, including the residual change variable and the moderator variables, without the interaction effect (Models 2a, 3a, 4a). Comparing the explained variance of the multiple regression models with the moderation analyses allowed us to test the effect sizes of the moderation analyses (ΔR^2^). For all models, we tested for linearity, normality of residuals, multicollinearity, homoscedasticity, and independence of errors. All assumptions were met, supporting the appropriateness of the moderation analysis.

## Results

### Descriptive results

Table 1 presents the intercorrelations among all measured constructs, the residual change variable from the regression of TSE-SRL at t1 on t2 (i.e., the development of TSE-SRL from t1 to t2), and the variable indicating teachers’ affiliation to either the CG or EG. All the measures showed acceptable to excellent internal consistency. T-tests revealed no significant baseline differences between the EG and CG for TSE-SRL (*t* = 0.24, *p* =.81), previous SRL experience (*t* = 0.91, *p* =.37), SRL mindsets (*t* = 0.53, *p* =.60), or teachers’ SRL skills (*t* = −0.11, *p* =.91).

The results indicate only weak to moderate correlations between the variables, with only low or marginal significance. However, the correlation between the group affiliation (coded to reflect the SRL-focused PD) and TSE-SRL development was significant (*r* = 0.27, *p* =.07), indicating that the EG showed higher TSE-SRL development. Further, teachers’ previous experience in promoting SRL showed a significant correlation with their initial TSE-SRL levels (*r* = 0.59, *p* <.01), and also with TSE-SRL at t2 (*r *= 0.29, *p* <.05), suggesting that prior experience may contribute to overall higher self-efficacy. However, it was not significantly related to changes over time. A similar pattern was found for teachers’ own SRL skills, which were positively associated with initial TSE-SRL (*r* = 0.31, *p* <.05), but not significantly with their development. These findings offer initial, descriptive support for the role of prior experience and PD focus on shaping teachers’ TSE-SRL.

### Effect of mastery and vicarious experiences focus on teachers’ self-efficacy

The means for TSE-SRL raised from t1 to t2 for the EG (*Mt1*_*EG*_ = 3.42, *Mt2*_*EG*_ = 4.07) and the CG (*Mt1*_*CG*_ = 3.48, *Mt2*_*CG*_ = 3.82) with slightly higher raises in the EG (Δ*M*_*EG*_ = 0.64, Δ*M*_*CG*_ = 0.34). According to Cohen (1988), this corresponds to a large effect (*d* = 0.73) in the EG and a small to moderate effect in the CG (*d* = 0.47). The overall group difference was small (*d* = 0.36). The results from the multiple regression model indicate that the overall model, predicting the development of TSE-SRL based on group affiliation (PD program focus) and teachers’ professional experience, was significant, *F*(2, 45) = 3.05, *p* =.028. Within this model, group affiliation significantly predicted the development of TSE-SRL (β =.28, *p* =.023), whereas professional experience did not emerge as a significant predictor. According to Cohen (1988), the explained variance for the model was *R*^2^ =.12, indicating a low to moderate goodness-of-fit.

### Effects of the moderating variables

**Fig. 3:**
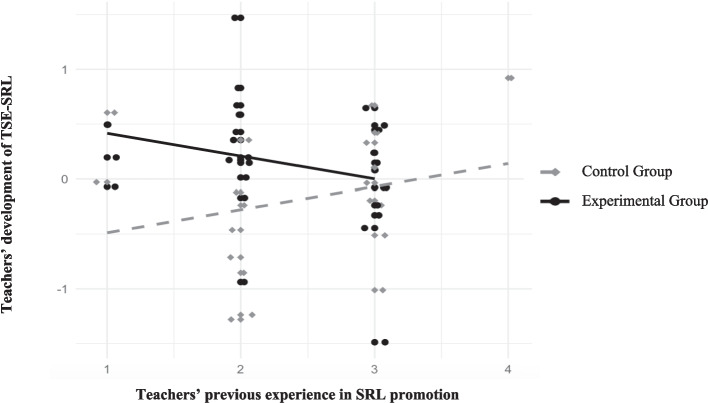
Moderation effect of teachers’ previous experience in promoting SRL on the relationship between PD focus and the development of TSE-SRL. The y-axis represents standardized residual change scores, calculated by regressing TSE-SRL at t2 on TSE-SRL at t1

Tables 2, 3, and 4 show the results of the models calculated to detect moderating effects. From the models, only teachers’ previous experience in promoting SRL reached the limit to significantly negatively moderate the effect of mastery and vicarious experiences within the PD program on TSE-SRL development (*F*(3, 44) = 2.18, *p* =.501) (see Table 2). Figure 3 shows that within the EG (solid black line), teachers with higher experiences showed less development of their TSE-SRL than teachers with fewer previous experiences (dashed grey line). At the same time, in the CG, this relation was inverse: Teachers with more experience in promoting SRL showed higher development of their TSE-SRL than teachers with less experience. The model 2a explained 5% more variance than the corresponding model 2b, including the two variables as predictor (see Table 2).

**Table 2:** Moderation effect of teachers’ previous experience in promoting SRL on the relationship between mastery and vicarious experiences focus and the development of TSE-SRL

Variables	*B*	β	SE	*p*	*B*	β	SE	*p*
	Model 2a				Model 2b			
Constant^a^	−0.16	-	0.33	0.309	−0.69	-	0.44	0.062
Mastery and vicarious experiences focus in PD program	0.31*	0.26*	0.17	0.036	1.32*	1.10*	0.60	0.017
Previous experience in promoting SRL	−0.01	−0.01	−0.12	0.481	0.20	0.24	0.17	0.116
Sources of self-efficacy*experience in SRL promotion					−0.41*	−0.88*	0.24	0.045
R^2^			0.07	0.192			0.12	0.051
ΔR^2^							0.05	

^*^p <.05. ^**^p <.01 (one-tailed). *B* = unstandardised regression coefficient; β = standardised regression coefficient; SE = standard error; *R*^*2*^ = explained variance; Δ*R*^*2*^ = change in explained variance compared to the model 2a

^a^ The constant represents the intercept of the regression model for the control group (coded as 0)

**Table 3:** Moderation effect of teachers’ SRL mindsets on the relationship between mastery and vicarious experiences focus and the development of TSE-SRL

Variables	*B*	β	SE	*p*	*B*	β	SE	*p*
	Model 3a				Model 3b			
Constant^a^	0.87	-	0.84	0.302	2.14	-	1.29	0.103
Mastery and vicarious experiences focus in PD program	0.29	0.24	0.17	0.085	−1.84	−1.54	1.67	0.274
SRL mindsets ^b^	−0.19	−0.18	0.15	0.210	−0.42	−0.39	0.23	0.076
Sources of self-efficacy*SRL mindset					0.39	1.79	0.30	0.203
R^2^			0.10	0.087			0.13	0.090
ΔR^2^							0.03	

^*^p <.05. ^**^ *p* <.01. *B* = unstandardised regression coefficient; β = standardised regression coefficient; *SE* = standard error; R^2^ = explained variance; ΔR^2^ = change in explained variance compared to the model 2a

^a^ The constant represents the intercept of the regression model for the control group (coded as 0)

^b^ Higher values in the mindset variable indicate a stronger growth mindset about SRL

**Table 4:** Moderation effect of teachers’ SRL skills on the relationship between mastery and vicarious experiences focus and the development of self-efficacy

Variables	*B*	β	SE	*p*	*B*	β	SE	*p*
	Model 4a				Model 4b			
Constant^a^	−0.90	-	0.72	0.223	−0.66	-	1.10	0.552
Mastery and vicarious experiences focus in PD program	0.32	0.27	0.17	0.061	−0.09	−0.07	1.46	0.951
SRL skills	0.15	0.14	0.15	0.320	0.10	0.09	0.23	0.661
Sources of self-efficacy*SRL skills					0.09	0.35	0.31	0.775
R^2^			0.06	0.117			0.09	0.228
ΔR^2^							0.03	

^*^p <.05. ^**^ *p* <.01; *B* = unstandardised regression coefficient; β = standardised regression coefficient; *SE* = standard error; R^2^ = explained variance; ΔR^2^ = change in explained variance compared to the model 2a

^a^ The constant represents the intercept of the regression model for the control group (coded as 0)

## Discussion

Various studies highlight the crucial role of TSE-SRL for teachers’ SRL promotion (e.g. Chatzistamatiou et al., 2014; Jud et al., 2023). Although the positive effect of interventions on TSE development exists (see Täschner et al., 2024), there are few and mixed results regarding the effect of PD programs on promoting TSE-SRL. Furthermore, previous research on the development of TSE-SRL has not explicitly referenced experiences as a source of self-efficacy, and there are almost no findings available on what moderates this development. This study expands prior research by investigating the effect of mastery and vicarious experiences as sources of self-efficacy within a PD program on teachers’ development of TSE-SRL. Additionally, the moderating effect of three different variables on this effect is analysed: Teachers’ previous experience in SRL promotion, their mindsets about SRL, and their own SRL skills.

### Effects of the mastery and vicarious experiences focus on teachers’ self-efficacy to promote SRL

As hypothesised (H1), the results from the regression model revealed a higher level of self-efficacy after the PD program, and the EG showed a marginally significant higher self-efficacy development than the CG. This confirms the prior finding that TSE can be successfully developed within PD programs (Täschner et al., 2024). This is an important finding as TSE is assumed to be domain-specific (Tschannen-Moran et al., 1998). Based on the means and standard deviations retrieved from other studies in the context of SRL, the effect sizes within the EG (*d* = 0.72) were higher than within the short-term intervention from Dignath et al. (2021) (*d* = 0.57) and in the comparable intervention from Heirweg et al. (2021) (*d* = 0.45). Regarding the effect sizes between the groups, our study showed higher effect sizes (*d* = 0.36) than the comparable long-term intervention (*d* = 0.06) but lower effects than the short-time intervention (*d* = 0.52) These comparisons show that the teachers in the EG of our study developed their TSE-SRL more than in other SRL PD programs. This indicates that explicitly including mastery and vicarious experiences into PD programs might be a promising approach to foster TSE-SRL, especially in the long term. However, the large effect size observed in the EG can be attributed not only to a greater mean increase in TSE-SRL, but also to a reduction in variance from pre- to post-measurement (*SD*_t1_ = 1.04; *SD*_t2_ = 0.67). This decrease in standard deviation indicates that the PD program appears to have supported not only overall growth in TSE-SRL, but also a convergence in teachers’ self-efficacy beliefs regarding SRL promotion.

The CG also showed a small to moderate improvement in TSE-SRL, and the significance of the effect between the groups was rather low. This may be due to overlapping elements between the two PD programs. In our study, the design and content of the alternative treatment of the CG might have been too close to the treatment of the EG, thus reducing the differences regarding teachers’ development of their TSE-SRL. Täschner et al.’s (2024) findings indicate that studies including an alternative treatment for the CG showed lower effects regarding the development of TSE than studies with no alternative treatment for the CG. Thus, the detected effect within this study might have been higher with a CG without treatment. These findings suggest that even non-SRL-specific PD programs may incidentally support SRL-related self-efficacy, particularly when they include components associated with mastery and vicarious experiences, and the content focus is not distinct enough. Therefore, a stronger, domain-specific focus appears necessary to produce larger and more consistent effects.

### Effects of the moderating variables

In RQ2, we examined individual factors, including teachers’ previous experiences in promoting SRL (RQ2a), teachers’ growth mindsets about SRL (RQ2b), and teachers’ SRL skills (RQ2c) that might moderate the effect of the PD program on the development of TSE-SRL. The following sections discuss the results.

#### The effect of teachers’ previous experience in promoting SRL

We expected that teachers’ previous experience in promoting SRL would negatively affect the effect of the mastery and vicarious experiences within the SRL PD program on TSE-SRL (H2a). Accordingly, teachers’ previous experiences negatively moderated the effect of the experiences within the PD program on the development of TSE-SRL. Teachers with lower reported experience at the beginning of the PD program showed a stronger development of their TSE-SRL. Thus, as proposed by Bandura (1997), the effect of mastery and vicarious experiences on TSE-SRL was stronger for teachers with less previous experience in promoting SRL. This effect might have been even stronger due to teachers’ misconceptions about SRL prior to the PD program. Teachers often conceptualise SRL as self-directed learning, such as students requiring minimal teacher support or self-pacing their work. This differs from the SRL processes described in research (Callan & Shim, 2019) and included in our PD program. Although self-directed learning can become a central part of indirect SRL promotion, it has to be adequately scaffolded and accompanied by direct promotion (Karlen & Hertel, 2024; Zimmerman, 2000). If teachers’ experiences regarding SRL primarily involved students working autonomously without explicit SRL instruction, they might have initially overestimated their ability to foster SRL. During the PD program, they may have realised that effective SRL promotion requires both direct and indirect SRL promotion (Karlen & Hertel, 2024; Zimmerman, 2000). This could have been particularly impactful for teachers with initially high TSE-SRL, as they might have faced a stronger discrepancy between their prior beliefs and the broader SRL framework presented in the PD program.

#### The effect of teachers’ SRL mindsets

We expected that teachers’ SRL mindsets would positively affect the effect of the mastery and vicarious experiences on TSE-SRL within the SRL PD program (H2b). Against this hypothesis, we did not find any significant effects of teachers’ SRL mindsets moderating the effect of mastery and vicarious experiences on TSE-SRL. First, the question arises as to which constructs and how teachers’ SRL mindsets are linked. A meta-study has revealed that teachers’ growth mindsets are linked to their TSE (Bardach et al., 2024). However, in the context of SRL, teachers’ growth mindsets may be more directly related to their perceived importance of promoting SRL, as shown in previous research, than to moderating the development of TSE-SRL (Karlen et al., 2023a). Further, previous results indicate that TSE mediates the effect of teachers’ mindsets on their classroom practices (Lüftenegger & Muth, 2024). This suggests that teachers’ mindsets may not act as moderator of TSE-SRL effects, but rather that TSE-SRL may be a mediator of the influence of teachers’ mindsets on their SRL promotion. More research would be needed to disentangle the reciprocal or potentially bidirectional links between teachers’ mindsets and their TSE-SRL.

Second, due to the lack of empirical evidence, we relied on theoretical assumptions to support our hypothesis. Mindsets might work as lenses through which teachers interpret information from their mastery and vicarious experiences (Chen & Usher, 2013; Eccles & Wigfield, 2020). However, mastery experiences are assumed to influence TSE only when teachers perceive them to be aligned with a certain activity (Morris et al., 2017). Thus, teachers must first recognise that their promotion influences students’ SRL skills before considering their own mindsets relevant for developing their TSE-SRL. However, research has shown that teachers have difficulties assessing their students’ SRL skills (Karlen et al., 2023a). Thus, teachers’ ability to assess their students’ SRL development might be more important than how their mindsets influence their way of interpreting students’ development. Additionally, our data do not provide information on whether teachers perceived their promotions as successful and whether these experiences were related to growth or fixed mindsets. Therefore, future research should focus in more detail on whether they perceive their promotion as a successful or unsuccessful experience, whether they relate this outcome to their ability to detect students’ development in SRL, and how their mindsets affect their TSE-SRL development in terms of their perception. In this regard, Ding et al. (2019) demonstrated that their attributional processes moderated the development of pre-service TSE during internships. Therefore, including actual attributional processes in the analysis (Weiner, 1985) could contribute to a better understanding of the chain of interpretation and the role of teachers’ mindsets.

#### The effect of teachers’ own SRL skills

We expected that teachers’ SRL skills would positively moderate the effect of mastery and vicarious experiences within the PD program on the development of TSE-SRL (H2c). Against this hypothesis, we found no significant moderating effect. First, this could be due to a low variance among the participants regarding the relatively high mean of their reported SRL skills at the beginning of the teacher training. Second, the PD program incorporated structured moments of group reflection within the EG. In these sessions, teachers reflected together on their implementation of SRL-related measures in their classrooms. This process can be understood as socially shared regulation of learning (SSRL) (Panadero & Järvelä, 2015), in which group members co-construct regulatory processes. In such settings, the individual level of SRL skills may have played a less prominent role, as regulatory processes were conducted in the reflection groups. This may explain why teachers’ own SRL skills did not moderate the effect of the SRL PD program. In contrast, in less structured or more individual PD programs, individual SRL skills might be more critical for successfully developing TSE-SRL. Third, our study focused on teachers’ SRL skills as they have been conceptualised as part of their professional competencies (Karlen et al., 2020). However, Kramarski and Heaysman (2021) also highlight the necessity of teachers’ competencies in regulating their teaching of SRL (SRT) alongside their own SRL skills. According to their model, SRT competencies are crucial for teachers to collect successful mastery experiences in promoting SRL. Therefore, on the one hand, teachers’ SRT competencies for SRL promotion may be more important in relation to their interpretative processes of experiences regarding SRL promotion and, thus, for developing their TSE-SRL. On the other hand, teachers’ own SRL skills might be more important for their SRL teaching. Empirical evidence exists that teachers’ own SRL skills are related to their self-efficacy and indirectly to their students’ SRL competencies (Karlen et al., 2024). Teachers’ own SRL skills might help them better understand the development of their students’ SRL skills or the difficulties they face during this process (Karlen et al., 2023a; Peeters et al., 2014). This also relates to general models of teachers’ professional competencies, showing that teachers’ knowledge, beliefs, and motivation are relevant for their teaching quality (Baumert & Kunter, 2013; Blömeke et al., 2015). However, more research is needed on the personal and contextual determinants of individual teacher development of professional competencies, especially in the field of SRL.

### Limitations

Several limitations must be considered when interpreting the results of this study. First, our study did not include measures to assess teachers’ mastery and vicarious experience directly. Therefore, the results must be interpreted cautiously, and no conclusions can be drawn about the relative strength of mastery and vicarious experiences in developing self-efficacy. Thus, for a better understanding of TSE-SRL, more sensitive measures should be applied to assess the different sources of self-efficacy, including mastery and vicarious experiences (see Morris et al., 2017). This also includes applying more experimental designs to systematically investigate the relative effect of the different self-efficacy sources on TSE-SRL or combinations of sources for teachers with different levels of experience in SRL promotion (Tschannen‐Moran & McMaster, 2009).

Second, this study relied on quantitative data based on teachers’ self-reports. However, research on the development of motivational constructs, such as self-efficacy, should also consider methodologies to gain more in-depth insights into the process rather than the relationships between constructs. For this reason, future studies could include interviews to assess how teachers evaluate and process information about the sources of self-efficacy.

Third, the study had a relatively small sample size. Therefore, effect sizes and significances must be interpreted with caution. A small sample size can reduce statistical power, increasing the likelihood of Type II errors (Cohen, 1988). Furthermore, generalising the results to different settings or groups is delicate (Maxwell, 2004). Accordingly, future studies analysing the effect of self-efficacy sources on the development of TSE-SRL and variables moderating that relationship should consider larger samples to validate the results of this study.

### Practical implications

The study's results can inform the design of future SRL PD programs. First, the results confirm that fostering teachers’ mastery and vicarious experiences in a PD program can effectively develop TSE-SRL, as shown in other educational contexts (Täschner et al., 2024). Therefore, the study underscores the relevance of explicitly integrating elements that support teachers’ mastery and vicarious experience into PD design. However, as we did not assess the effect of the sources separately, it is impossible to draw any practical conclusions about the relative importance of mastery and vicarious experiences. Considering other studies, focusing mainly on mastery experiences might be important when not all sources can be incorporated (Täschner et al., 2024).

Second, the study also highlights the importance of considering the heterogeneity of teacher backgrounds and tailoring PD components accordingly. According to the previous level of SRL experience, teachers might need different measures to develop their TSE-SRL, such as individual support activities. For instance, previous studies have shown that such individual support activities might strengthen the effect of the sources of self-efficacy (Mok et al., 2023). Finally, although teachers’ own SRL skills did not moderate the TSE-SRL development, the theoretical discussions in the study indicate that teachers’ own SRL skills might be important in less structured PD settings.

## Conclusion

In our study, we tested the hypothesis that TSE-SRL can be developed through mastery and vicarious experiences within a PD program. Furthermore, we hypothesised that this development would be negatively moderated by teachers’ level of experience in promoting SRL and positively moderated by their SRL mindsets and their own SRL skills. The results showed that TSE-SRL was developed through the PD program in both groups. However, the EG demonstrated a slightly higher improvement than the CG. Further, only teachers’ previous experiences moderated this development as expected. The conclusion from these results, along with the comparison with other studies, is that future interventions should consider the design of PD programs in relation to teachers’ experience levels and include measures to promote TSE-SRL in accordance with their experience levels in promoting SRL. However, future studies should investigate the contribution of different sources of self-efficacy more specifically. This includes teachers’ perception of their students’ SRL skill level and development, and how they interpret this development (attributional styles). Overall, more specific and qualitative measures regarding the sources of self-efficacy are needed to understand the development of TSE-SRL.

## Data Availability

Data will be made available on request. The R scripts used for data analysis are available at https://osf.io/ar6pu/?view_only=f33cabac7beb4727903030421c8e9cca.
